# Editorial: Application of data mining in pharmaceutical research

**DOI:** 10.3389/fphar.2024.1388738

**Published:** 2024-03-01

**Authors:** Qingting Bu, Jun Lyu, Limei Zhao, Shiyi Cao, Deyong Jia, Zhenyu Pan

**Affiliations:** ^1^ Department of Genetics, Northwest Women’s and Children’s Hospital, Xi’an, Shaanxi, China; ^2^ Department of Clinical Research, The First Affiliated Hospital of Jinan University, Guangzhou, Guangdong, China; ^3^ Department of Pathology and Pathophysiology, Sochoow University, Suzhou, Jiangsu, China; ^4^ School of Public Health, Tongji Medical College, Huazhong University of Science and Technology, Wuhan, Hubei, China; ^5^ Department of Urology, University of Washington, Seattle, WA, United States; ^6^ Department of Pharmacy, Xi’an Children’s Hospital, Xi’an, Shaanxi, China

**Keywords:** big data, data mining, database, pharmaceutical research, health

Big data from medical public databases and data-mining techniques are pivotal to advancing various healthcare fields, particularly epidemiology, public health, and clinical research. These databases provide a wealth of information on health indicators, disease prevalence, and patient outcomes, and applying data-mining techniques to them can reveal trends, correlations, and patterns for informing public health policies and clinical practices (Yang et al., 2020; Wu et al., 2021). For example, researchers can analyze data on disease incidence and mortality rates to identify risk factors and develop targeted interventions for reducing disease burdens. In clinical research, mining data obtained from medical databases aids in identifying potential drug targets, evaluating treatment efficacy, and predicting patient outcomes. This can lead to the development of new therapies and the optimization of existing treatments, ultimately improving patient care and outcomes. However, relatively few studies have applied data-mining techniques to various medical databases with a focus on pharmacy. Therefore, this Research Topic focuses on the application of data mining in pharmaceutical research. Below we introduce and comment on the 11 research articles comprising this Research Topic.

Chen et al. investigated the relationships of low- and high-dose aspirin use with the risks of death from all causes, cardiovascular disease (CVD), and cancer among US adults aged 40 years and older using data from the NHANES (National Health and Nutrition Examination Survey). The study found that low-dose aspirin did not significantly reduce the risk of death from any cause, whereas high-dose aspirin use was associated with an increased risk of CVD death. The study highlights the need for caution in recommending high-dose aspirin for the primary prevention of CVD, especially in older adults, due to the potential increased risk of adverse outcomes.

Fang et al. conducted a real-world disproportionality analysis of apalutamide, a drug used to treat prostate cancer, using data from the FDA Adverse Event Reporting System. The analysis aimed to identify the safety of apalutamide in real-world settings, and yielded valuable evidence for the safety profile of apalutamide in clinical practice. Wang et al. conducted a retrospective pharmacovigilance analysis using the same database to identify drugs associated with tooth discoloration. The authors concluded that caution is needed when using these drugs, especially during pregnancy and early childhood. However, they also stated that further investigations are needed to confirm their findings and fully understand the mechanisms underlying tooth discoloration.

Li et al. analyzed the adverse drug reactions (ADRs) of five anti-TNFα agents using data from the WHO-VigiAccess database. The analysis identified common ADRs and their proportion. The findings suggest the importance of monitoring and the rational use of these drugs due to their potential for serious ADRs.

Del Fiol et al. analyzed the sales trends of psychotropic drugs in Brazil during the COVID-19 pandemic in order to identify any changes in consumption patterns. Using data from the National System of Controlled Products Management, the researchers found that sales of certain psychotropics increased significantly during the pandemic. The findings suggest that the pandemic has had a significant negative impact on mental health that has led to an increased use of these medications.

Three articles by Zhu et al., Wu et al., and Yang et al. used the Medical Information Mart for Intensive Care database. Zhu et al. aimed to determine the association between glucocorticoid use and all-cause mortality in critically ill patients with heart failure. The authors recommended that glucocorticoids should not be given to critically ill patients with heart failure, but also stated that further prospective studies or randomized controlled trials are needed for validation. Wu et al. analyzed the impact of ondansetron on acute pancreatitis patients in the ICU, and produced findings supporting the use of ondansetron as an antiemetic in ICU patients with acute pancreatitis. Yang et al. investigated the association between thiamine administration and prognosis in critically ill patients with heart failure, with the findings suggesting that thiamine supplementation can improve the prognosis of critically ill patients with heart failure.

Bu et al. analyzed global trends in the incidence rates of multidrug-resistant (MDR) and extensively drug-resistant (XDR) tuberculosis (TB) from 2010 to 2019 using data from the Global Burden of Disease Study 2019. The study found that the age-standardized incidence rate (ASR) of MDR-TB decreased over the analyzed period, while that of XDR-TB remained stable. The incidence trends varied by region, with most regions showing a decrease in MDR-TB but an increase in XDR-TB. People aged 35–44 and 55–64 years had the highest incidence rates of both MDR-TB and XDR-TB. Regions with a higher sociodemographic index (SDI) tended to have lower ASRs of MDR-TB. The authors concluded that current efforts to curb MDR-TB and XDR-TB are insufficient, and hence new strategies are needed, especially in high-risk regions and age groups as well as low-SDI regions.

Zhang et al. analyzed the risk factors and developed predictive models for acute kidney injury (AKI) in hospitalized patients treated with cefoperazone-sulbactam sodium and mezlocillin-sulbactam sodium using multivariate logistic regression analysis. The findings suggest that evaluating risk factors before administering these antibiotics can reduce the incidence of AKI and emphasize the need for increased awareness among medical staff and patients regarding AKI.

Luo et al. developed and validated a nomogram for predicting pulmonary infection (PI) in patients receiving immunosuppressive drugs based on the LASSO (least absolute shrinkage and selection operator) and multivariate Cox regression analyses. The study concluded that the nomogram could aid in predicting the PI risk in individual patients receiving immunosuppressive treatment and assist in personalized clinical decision-making. This suggests that the nomogram model has potential for widespread implementation in clinical practice to reduce the occurrence of PI.

In summary, the application of big data and data mining in pharmaceutical research supplements and adds practical evidence that can support decision-makers and clinicians. This Research Topic presents recent evidence and new perspectives for this.
